# Improving Wear Resistance and Corrosive Resistance of Cemented Carbide for Mud Pulser Rotor by Deep Cryogenic Treatment

**DOI:** 10.3390/ma17051195

**Published:** 2024-03-04

**Authors:** Weiguo Zhang, Xiaowei Wu, Jun Tian, Xi Huang, Wentao Yu, Wenchao Zhu, Jingwen He

**Affiliations:** 1 School of Environment and Civil Engineering, Dongguan University of Technology, Dongguan 523808, China; tianjun@dgut.edu.cn (J.T.); huangxibeyond@163.com (X.H.); yuwentaobridge@163.com (W.Y.); zhuwenchaogg66@163.com (W.Z.); hejingwenbridge@163.com (J.H.); 2 Guangdong Provincial Key Laboratory of Intelligent Disaster Prevention and Emergency Technologies for Urban Lifeline Engineering, Dongguan University of Technology, Dongguan 523808, China; 3 College of Construction Engineering, Jilin University, Changchun 130026, China

**Keywords:** cemented carbide, deep cryogenic treatment, wear resistance, electrochemical corrosion, microstructure

## Abstract

Cemented carbide used in the rotor of a mud pulser is subjected to the scouring action of solid particles and corrosive mud media for a long time, which causes abrasive wear and electrochemical corrosion. To improve the wear and corrosive resistance of cemented carbide, samples with different cobalt content (WC-5Co, WC-8Co, and WC-10Co) receive deep cryogenic treatment (DCT) at −196 °C for 2.5 h. An optical metalloscope (OM) and X-ray diffractometer (XRD) are used to observe the phase changes of cemented carbides, and the XRD is also used to observe the change in residual stress on the cemented carbide’s surface. A scanning electron microscope (SEM) is used to characterize the wear and electrochemical corrosion surface microstructure of cemented carbides (untreated and DCT). The results show that the DCT promotes the precipitation of the η phase, and the diffraction peak of ε-Co tends to intensify. Compared with the untreated, the wear rates of WC-5Co, WC-8Co, and WC-10Co can be reduced by 14.71%, 37.25%, and 41.01% by DCT, respectively. The wear form of the cemented carbides is mainly the extrusion deformation of Co and WC shedding. The precipitation of the η phase and the increase in WC residual compressive stress by DCT are the main reasons for the improvement of wear resistance. The electrochemical corrosion characteristic is the dissolution of the Co phase. DCT causes the corrosion potential of cemented carbide to shift forward and the corrosion current density to decrease. The enhancement of the corrosion resistance of cemented carbide caused by DCT is due to the Co phase transition, η phase precipitation, and the increase in the compressive stress of cemented carbide.

## 1. Introduction

The mud pulser is a measurement while drilling (MWD) instrument which can realize the real-time transmission of downhole signals. The rotor of the mud pulser is usually made of cemented carbide. Under the scouring action of mud containing solid particles and corrosive media, the rotor will produce wear and electrochemical corrosion, which attenuate the strength of the mud pulse signal and lead to the failure of signal decoding. If the rotor breaks due to wear and electrochemical corrosion, the rotor debris, driven by mud, will damage the equipment. If the rotor is replaced, it will need to lift the drill after a period of work, resulting in a waste of manpower and financial resources [1,2]. The wear and electrochemical corrosion of rotors can be reduced by optimizing rotor structure design. However, the rotor is always in contact with mud during rotation, which can reduce the abrasive wear and electrochemical corrosion of the rotor in a short period of time. Ultimately, the rotor failure is caused by the insufficient performance of cemented carbide material [3]. Cemented carbides used in rotors have been subjected to wear and corrosive media for a long time, so higher requirements for the wear resistance and corrosion resistance of cemented carbides have been put forward.

Studies have found that changing the material formula [4,5], laser cladding, and coating reinforced surfaces [6,7] can all improve the wear resistance of cemented carbides. However, the above methods involve many factors such as equipment, technology, cost, and effect, resulting in a certain degree of restrictions on the implementation. DCT is a kind of post-treatment technology that has attracted much attention in recent years. As an environment-friendly heat treatment process, DCT has the characteristics of simplicity, economic, and high efficiency, which can improve the life of parts at the lowest cost.

Li et al. [8] carried out a study on the DCT of cemented carbide tools, and the study showed that DCT refined WC particles and improved the hardness of the WC-Co matrix. DCT has a significant effect on the internal stress of the material, and the internal stress can be alleviated or even eliminated through cryogenic treatment; on the contrary, the residual stress in the material can also be increased [9,10,11,12]. Zhang et al. [13] studied the phase structure of cobalt. The ε-Co volume fraction of the WC-Co cemented carbides increased after DCT, and the DCT could precipitate fine η phase grains to form a compact and stable matrix. In the study on the strengthening mechanism of DCT on WC-Co cemented carbides, researchers found that the DCT of WC-Co cemented carbides can inhibit the transition from α-Co(hcp) to ε-Co(fcc) at high temperatures; thus, obtaining higher strength and DCT at room temperature can also improve the strength and toughness of materials [14,15,16]. Kalsi et al. [17] showed that DCT of cemented carbide leads to better wear resistance. Abbas [18], Xie [19] and Padmakumar [20] observed that the hardness and wear resistance of cemented carbides were improved after DCT. 

To improve the corrosion resistance of cemented carbides, Zhu et al. [21]. explored the influence of WC grain size on the corrosion resistance of cemented carbides, and found that with the decrease in WC particle size, the corrosion resistance of cemented carbides in acidic media gets worse and worse. Surface coating technology can prevent electrochemical corrosion on the surface of cemented carbides and improve the corrosion resistance of cemented carbides. In alkaline media, the WC phase is the most prone to corrosion, while in acidic media, the corrosion trend of the Co-based binder phase is greater [22,23,24]. To improve the composition of the binder or add some alloying elements or their carbides in the binder Co, such as CrC, VC, TiC, TaC, Ru, Al, etc., they will form intermetallic compounds with the binder, thus improving the corrosion resistance of cemented carbides [25,26,27]. The bonding phase Co in WC-Co cemented carbide is prone to corrosion in corrosive media, which makes the hard-phase skeleton lose its bonding effect and become loose, thus accelerating wearing and resulting in material failure. Therefore, improving the corrosion resistance of the binder is an important method to improve the corrosion resistance of WC-Co cemented carbides. DCT can improve the residual compressive stress on the surface of cemented carbides and reduce the tensile stress of Co, which has a positive effect on reducing the stress corrosion of Co. However, there are few reports on the corrosion resistance of cemented carbides by DCT.

In this paper, the rotor-used cemented carbides (WC-5Co, WC-8Co, and WC-10Co) were DCT, and the influence rules of DCT on the wear resistance and corrosion resistance of cemented carbide with different Co contents were analyzed. By means of optical microscope (OM), X-ray diffraction (XRD), and scanning electron microscope (SEM), the microstructure of cemented carbide was observed, and the change in residual stress on the WC surface was calculated. At the same time, the wear and corrosion characteristics of cemented carbide were analyzed, and the strengthening mechanism of DCT on the wear and corrosion resistance of cemented carbide was revealed.

## 2. Materials and Methods

### 2.1. Specimen Preparation

The cemented carbides (WC-5Co, WC-8Co, and WC-10Co) for mud pulser rotor were produced by Zigong Cemented Carbide Co., Ltd. (Zigong, China). Cemented carbide is prepared by powder metallurgy, and the powder of each raw material is prepared through the process of batching, mixing, then drying and sintering. The raw materials mainly include tungsten carbide powder (WC), cobalt powder (Co), grain inhibitor (VC and TaC), and the basic parameters of each powder are shown in Table 1.

According to the composition of cemented carbide, the quantitative powder materials were weighed sequentially using an electronic balance, and the original powder was mixed by ball milling method. When ball milling, anhydrous ethanol was chosen as the medium, and the powder and WC balls with a diameter of 6 mm were put into a ball milling jar and mixed in a planetary ball mill (JX-4G, Shanghai Jingxin Industrial Development Co., Ltd., Shanghai, China), with a mass ratio of balls to material of 8:1, and a rotational speed of 250 r/min. After mixing, the powder was put into a vacuum-drying box and dried at a temperature of 80 °C. The powder was mixed in a vacuum-drying box. After mixing, the powder was dried in a vacuum-drying oven at 80 °C. After drying, the powder was sieved through a 100-mesh sieve and encapsulated.

**Figure 1:**
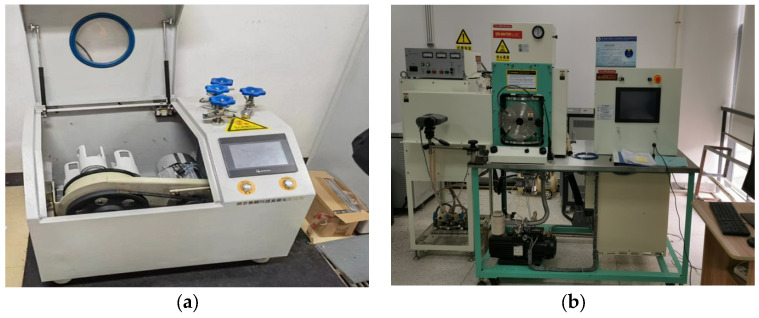
The equipment of sample preparation. (**a**) Planetary ball mill (JX-4G); (**b**) Plasma sintering furnace (SPS-1050T).

Weighing a predetermined mass of composite powder into a graphite mold for pre-pressing, the pressure is 30 MPa, time is 1 min, and then the graphite mold is placed into the plasma sintering furnace (SPS-1050T, Sumitomo Metal Mining Co., Tokyo, Japan) for sintering; the maximum sintering temperature of the furnace is 1700 °C. The ball mill and plasma sintering furnace are shown in Figure 1.

During the sintering process, the sintering temperature was controlled in the range of 1200–1350 °C, and an experimental temperature was set every 50 °C to study the optimum sintering temperature of cemented carbide. The temperature-increase rate was set at 100 °C/min, and the holding time after reaching the sintering temperature was 5 min.

**Figure 2:**
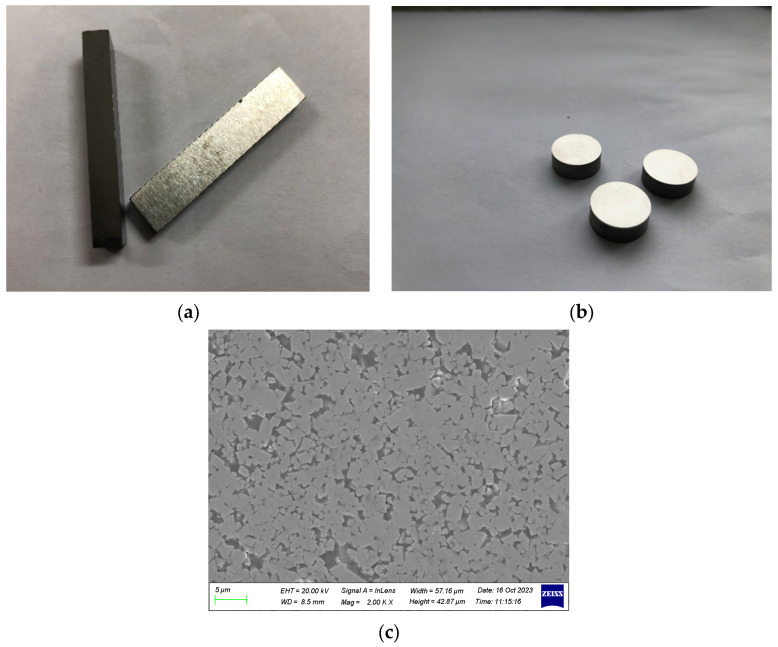
Samples of cemented carbide. (**a**) for wear resistance measurement (38 × 10 × 5 mm^3^); (**b**) for electrochemical corrosion measurement (Φ13 × 5 mm^3^); (**c**) the SEM image of cemented carbide.

Among them, the size of the sample for wear resistance measurement is 38 × 10 × 5 mm^3^, and for electrochemical corrosion measurement is Φ13 × 5 mm^3^; the specimens after sintering were firstly polished with 30 μm, 15 μm, 9 μm, and 3 μm diamond sandpaper, and then polished to a mirror-like surface with 1 μm diamond abrasive paste. An ultrasonic cleaner (DS-5510DTH, Shenzhen Fuyang Technology Group Co., Ltd., Shenzhen, China) was used to clean the surface of the specimens, which were placed in a vacuum-drying oven (DZF-6020, Shanghai Yiheng Scientific Instrument Co., Ltd., Shanghai, China) at 105 °C for 8 h and then removed. The polished cemented carbide sample and its microstructure are shown in Figure 2.

### 2.2. Methods

#### 2.2.1. DCT Process

A program-controlled low-temperature treatment chamber (SLX-250, Wuxi AISke Instrument Co., Ltd., Wuxi, China) is used for DCT experiments of cemented carbide: the adjustable temperature range of the device is from −196 °C to 190 °C, the cooling rate is from 0 °C/min to 60 °C/min, and the temperature control accuracy is ±2 °C. The DCT process of cemented carbide was divided into cooling stage, heat preservation stage, and temperature-rise stage.

**Figure 3:**
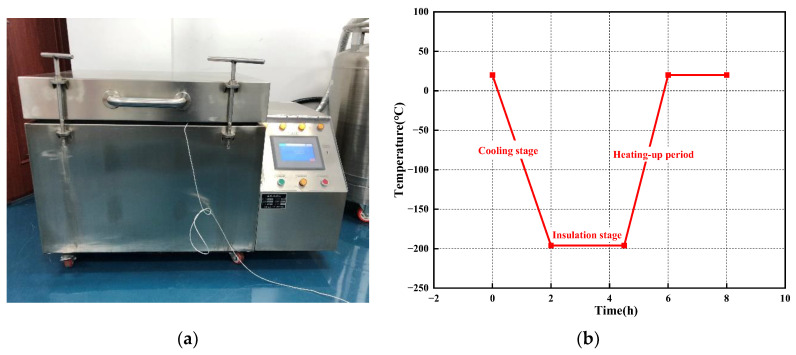
Deep cryogenic treatment device and DCT process. (**a**) SLX-250; (**b**) DCT process.

In this experiment, the temperature decreased from room temperature (about 20 °C) to −196 °C in 2 h (the cooling rate is 1.8 °C/min). The holding time was 2.5 h and then temperature rises from −196 °C to room temperature in 2 h (the heating rate is 1.8 °C/min). The device and process of DCT are as shown in Figure 3.

#### 2.2.2. Hardness

A Vickers hardness tester (MN-9631-130-C, INSIZE, Tianjin, China) was used to measure the hardness of the cemented carbide, the measurements were repeated at least five times, and the average value was calculated. The load selected for the measurement was 30 kgf, and the load-holding time was 15 s. Referring to the national standard GB/T 7997-2014 [28], the Vickers hardness was calculated as shown in Equations (1) and (2).

(1) $$H\nu =\frac{0.1891G}{D^{2}}$$

(2) $$D=\frac{D_{1}+D_{2}}{2}$$

where: Hν is Vickers hardness (MPa); G is the pressure (N); D is the average diagonal length of the indentation (mm).

#### 2.2.3. Wear Resistance Measurement

**Figure 4:**
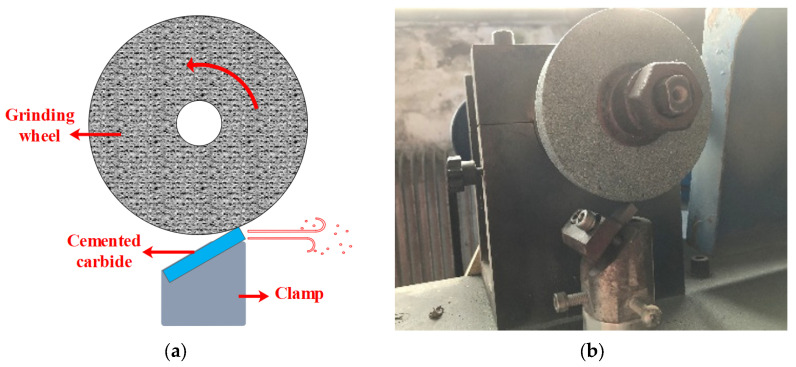
Abrasion resistance test of cemented carbide. (**a**) the principles of wear tester; (**b**) wear tester.

As shown in Figure 4, the wear resistance of cemented carbide is tested by wear tester (JS71-A, Tianjin Jinliang Instrument Equipment Co., Ltd., Tianjin, China), which consists of three parts: motor, grinding wheel, and fixture. During the test, the grinding wheel (GC80 × 16 × 20, Zhengzhou Hengfeng abrasives Co., Ltd., Zhengzhou, China) and the cemented carbides are ground against each other, the main material of the grinding wheel is SiC particles with a particle size of 80 mesh, and the wear rate of the cemented carbide is calculated through the recording of the wear mass loss of the cemented carbide. The test parameters are shown in Table 2.

After that, samples were cleaned in ethanol and weighed by an electronic balance with an accuracy of 0.1 mg. The mass loss of the sample after the wear experiment was used to characterize the wear resistance. The weight loss was measured every 30 s, the total test time was 300 s, each group tested three samples to ensure repeatability of the data.

The weight loss method is used to calculate the wear rate of cemented carbide, as shown in Equation (3), where $m_{1}$ is the weight before wear, $m_{2}$ is the weight before wear, and $W_{r}$ is the wear rate.

(3) $$W_{r}=\frac{m_{1}-m_{2}}{m_{1}}\times 100\%$$

#### 2.2.4. Electrochemical Corrosion Measurement

A customized fixture was used to completely close off the non-test surface of the cemented carbide, leaving only the polished surface as the test surface.

**Figure 5:**
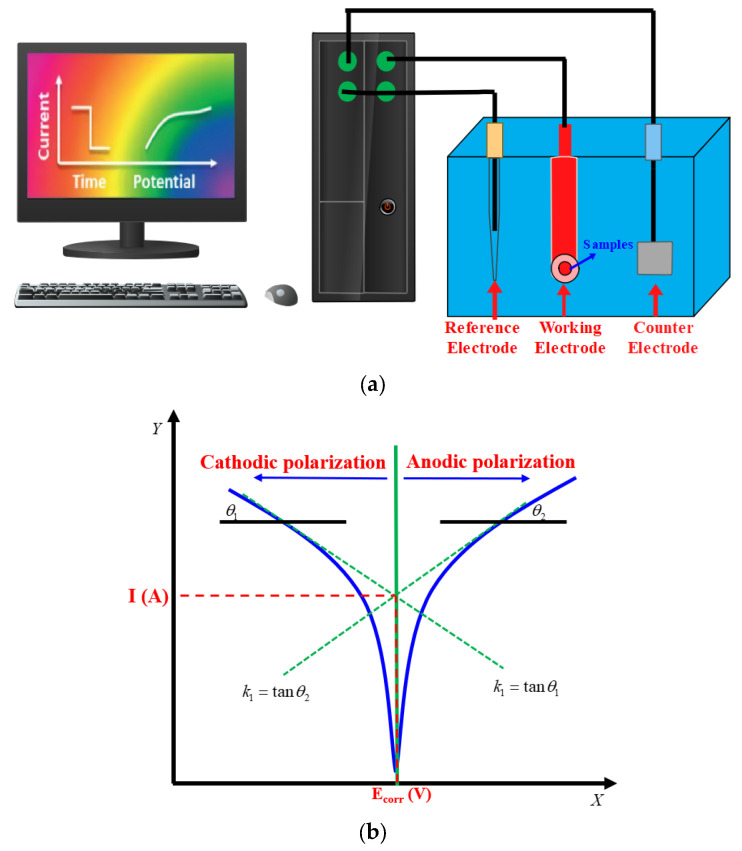
The test of electrochemical corrosion. (**a**) the principles of electrochemical corrosion experiment; (**b**) the principle of Tafel.

An electrochemical workstation (VersaSTAT 4, AMETEK, Berwyn, PA, USA) was used to test the polarization curves of cemented carbide, which is equipped with CS-Studio analysis software, capable of calculating important corrosion parameters such as corrosion potential, corrosion current density, etc. The electrochemical corrosion test principle is shown in Figure 5a: the three-electrode system is composed of working electrode, reference electrode, and counter electrode; in this study, the reference electrode is saturated calomel electrode (SCE), the counter electrode is platinum sheet, and the working electrode is a cemented carbide specimen. The corrosion medium used in the experiment was saturated NaCl solution (3.5 wt% NaCl), which was prepared from deionized water and NaCl.

In electrochemical corrosion experiments, the open-circuit potential was monitored for 1 h to obtain a stable open-circuit potential. Then, the specimens were subjected to dynamic potential scanning test to obtain the polarization curves, which can reflect the corrosion kinetic properties of the materials, in which the scanning potential of the dynamic potential polarization test is −1~1.5 V (vs SCE), and the scanning rate is 2 mV/s. The linear polarization region of the polarization curves was fitted using the analytical software, CS studio5.0 software, to obtain the corresponding parameters such as corrosion potential (E_corr_) and corrosion current density (i_corr_). The corresponding parameters of corrosion potential (E_corr_) and corrosion current density (i_corr_) were obtained. The fitting principle is shown in Figure 5b.

#### 2.2.5. Microstructure Characterization

The distribution of η phase of cemented carbide was observed by metallographic microscope. The microstructures of the worn surface and electrochemical corrosion surface of cemented carbide samples were characterized by scanning electron microscope (SEM, JSM-IT500A, JEOL, Tokyo, Japan).

X-ray diffraction (XRD, DX-2700BH, Dandong Haoyuan Instruments Co., Ltd., Dandong, China, Cu-Kα radiation, 40 kV, 30 Ma) was used to observe the phase changes of cemented carbides. The scanning speed was 2° every minute, and the step size was 0.02°. The scanning angle is from 30° to 90°. The change in residual stress on cemented carbide surface was collected by sin^2^ψ method. With the WC (300) crystal plane as the diffraction plane, the scanning angle range is 116°–119°, and the Ψ angle is 0°, 9°, 19°, 29°, 39°, 49°.

## 3. Results and Discussions

### 3.1. Metallographic and XRD

The η phase is the W, Co, C ternary compound formed by part of the Co atoms in the cooling process after sintering of cemented carbides. DCT provides the potential to further promote the formation of the η phase [29,30].

**Figure 6:**
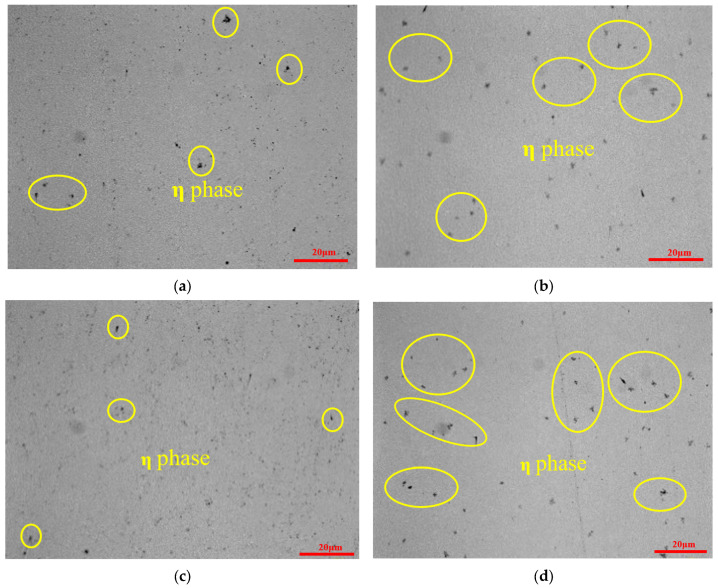
The metallographic diagram of η phase distribution of cemented carbide with untreated and DCT. (**a**) the η-phase distribution of WC-5Co with untreated; (**b**) the η-phase distribution of WC-5Co with DCT; (**c**) the η-phase distribution of untreated WC-10Co; (**d**) the η-phase distribution of WC-10Co with DCT.

Figure 6a–d show the changes in the metallographic structure of WC-5Co and WC-10Co cemented carbides (untreated and DCT). The η-phase is a compound of W, C, and Co, which appears as a gray–black spot under the metallographic microscope. As shown in Figure 6a,c, there was little η phase in untreated cemented carbides. But, as shown in Figure 6b,d, the η phase precipitates significantly and disperses uniformly in the form of dot blocks in DCT cemented carbides. The presence of the η phase has a dispersion-strengthening effect on the cemented carbide matrix and improves the properties of cemented carbide.

During the cooling process of WC-Co cemented carbides after sintering, the different thermal shrinkage between WC phase and Co phase causes internal stress in the material, and the “pinning” effect is generated by the solid solution of W and C in α-Co, thus inhibiting the crystal transformation from α-Co to ε-Co, leading to the stable existence of α-Co [31,32]. DCT eliminates the internal stress, which makes it possible to rearrange α-Co into a relatively stable, tightly-arranged hexagonal ε-Co structure.

**Figure 7:**
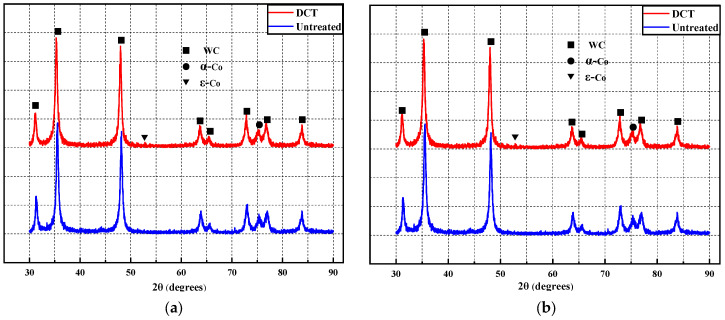
The XRD analysis of WC-Co with untreated and DCT. (**a**) the XRD analysis of WC-5Co with untreated and DCT; (**b**) the XRD analysis of WC-10Co with untreated and DCT.

Figure 7a,b shows the XRD patterns of WC-5Co and WC-10Co (untreated and DCT). As can be seen from Figure 7, the WC diffraction peak was more obvious after DCT, while the Co diffraction peak was not obvious, which may be due to the high content of WC and relatively low content of Co in the matrix. The diffraction peak of WC blocks the diffraction peak of Co. However, the diffraction peak of ε-Co tends to increase, which is due to the DCT promoting the crystal transformation from α-Co to ε-Co.

The results of residual compressive stress on the surface of WC-5Co, WC-8Co, and WC-10Co (untreated and DCT) are shown in Table 3. DCT can significantly increase the residual compressive stress of WC in cemented carbides; this is because when WC-Co receives deep cryogenic treatment, the low-temperature environment produces compressive and tensile stresses on WC and Co, and the resulting residual stresses increase the overall hardness and improve the wear resistance [33,34,35] (and, at the same time, the presence of residual stresses promotes the transition from α-Co to ε-Co, which has a higher corrosion resistance compared to α-Co [36,37,38,39]). In this experiment, the residual compressive stress of the WC surface in WC-5Co, WC-8Co, and WC-10Co increases by 84.11%, 91.38%, and 65.03%, respectively.

### 3.2. Wear Resistance

**Figure 8:**
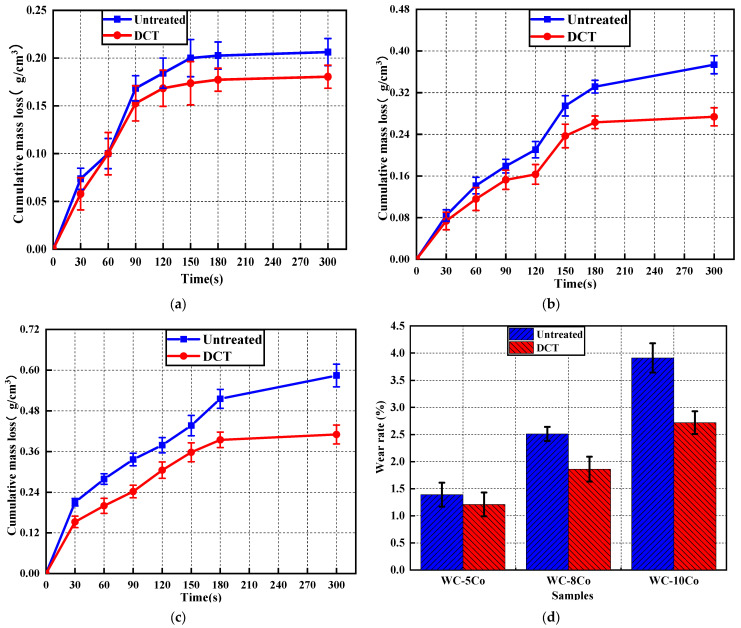
The wear–loss curve and wear rate of cemented carbide with untreated and DCT. (**a**) the wear–loss curve of WC-5Co; (**b**) the wear–loss curve of WC-8Co; (**c**) the wear–loss curve of WC-10Co; (**d**) the wear rate of cemented carbide.

Figure 8 shows the cumulative mass loss curves of WC-5Co, WC-8Co, and WC-10Co with different cobalt contents at each stage. As can be seen from Figure 8a–c, each curve had three wear stages. The first stage was from 0 to 30 s: at this time, the mass wear of the alloy was obvious; the mass loss of WC-10Co was the largest. The second stage is from 30 to 180 s: the mass loss of the cemented carbide was significantly different from that of the first stage. The mass loss of WC-5Co decreases obviously, and the mass loss of WC-10Co was always increasing. During the third stage, the mass loss of WC-5Co, WC-8Co, and WC-10Co increased from 180 to 300 s. Due to the increase in wear time, after partial removal of Co, WC particles were also dislodged due to the accumulation of friction damage, which led to a further increase in wear mass loss. However, the wear resistance of WC is better than that of Co, and the increase in Co content implies that the content of WC particles decreases, which in turn increases the loss of wear mass: it can be seen from the weight loss curve of WC-5Co, WC-8Co, and WC-10Co that the wear mass loss in cemented carbide was positively correlated with Co content.

As shown in Figure 8d, the DCT can reduce the wear rate of cemented carbide; the wear rate of WC-5Co, WC-8Co, and WC-10Co can be reduced by 14.71%, 37.25%, and 41.01% by DCT, respectively. The strengthening effect of DCT on different Co contents of cemented carbide is different: the higher the Co content in cemented carbide, the more obvious the strengthening effect, because Co in cemented carbide is the main removal phase of wear, and DCT will promote the transformation of α-Co to ε-Co, which has a better wear resistance compared to α-Co, and the DCT will increase the tensile stress of Co, which will increase the difficulty of Co removal by friction [36,37,38,39].

**Figure 9:**
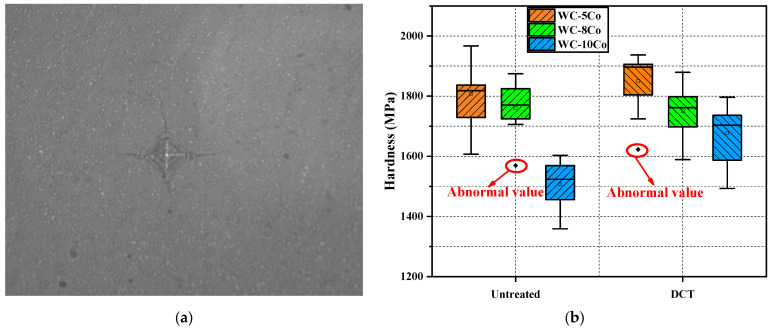
The indentation and Vickers hardness of cemented carbide. (**a**) the indentation of cemented carbide; (**b**) the Vickers hardness of cemented carbide.

The wear resistance of cemented carbide is directly related to the hardness, and to investigate the strengthening mechanism of DCT on the wear resistance of cemented carbide, the indentation and Vickers hardness of WC-5Co, WC-8Co, and WC-10Co was tested as shown in Figure 9. The DCT improved the hardness of cemented carbide, and the hardness was negatively correlated with the amount of wear. Because the increase in hardness increases the densification of the surface of the cemented carbide, the greater the resistance to the intrusion of SiC abrasives, the lower the possibility of microcracks, and the higher the wear resistance of the cemented carbide.

**Figure 10:**
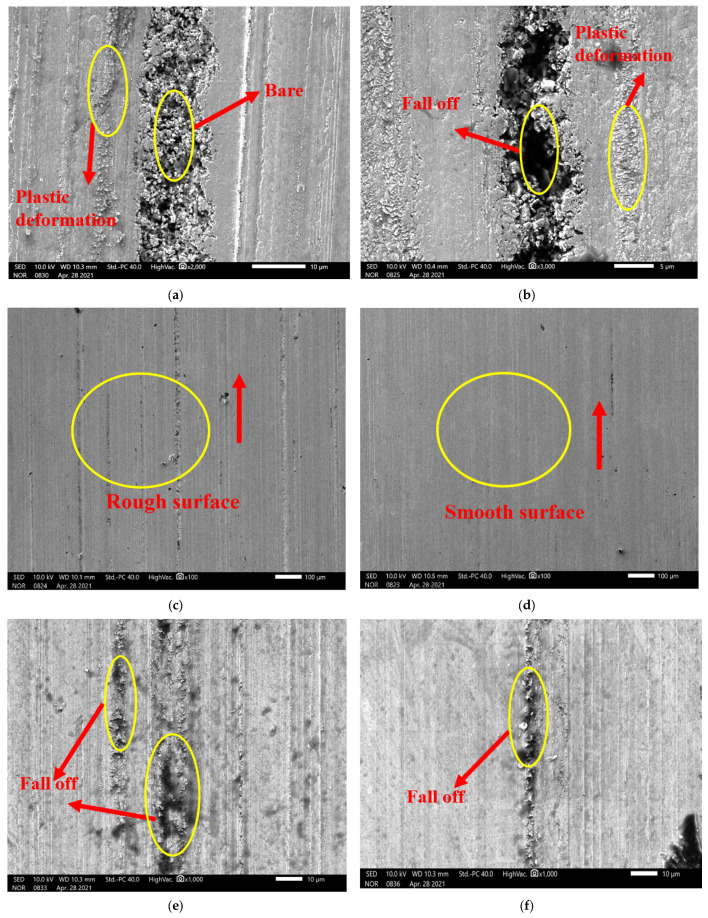
The SEM images of wear resistance of cemented carbide with untreated and DCT. (**a**) the WC particles exposed in cemented carbide; (**b**) the WC particles fall off from the cemented carbide; (**c**) the wear morphology of untreated cemented carbide; (**d**) the wear morphology of cemented carbide with DCT; (**e**) the wear morphology of untreated cemented carbide; (**f**) the wear morphology of cemented carbide with DCT.

Figure 10 shows the SEM image of worn surfaces of cemented carbide at different stages. It can be seen from Figure 10a that at the early stage of wear, groove marks were generated on the surface, and there was a certain plastic deformation near the groove marks. It shows that with the wear process, the Co bonding phase near the grooves had obvious extrusion deformation; when a certain amount of deformation was reached, the WC particles began to fall off a little because of the plastic deformation of the binder cobalt, which meant the hard-phase WC particles inside were exposed. The main wear mechanism in this stage was the deformation of the binding phase and the shedding of the hard-phase WC. As the wear of cemented carbide increases greatly, the extrusion deformation of binder Co and the shedding of WC particles of cemented carbide further increase. It can be seen in Figure 10b that large numbers of WC particles were shed in the groove marks, and the depth of the groove marks further increased. The main wear mechanism at this stage was the shedding of the hard phase.

It can be seen from Figure 10c,d that the wear surface of samples treated by DCT was smoother than that of samples without DCT at low magnification rates. As can be seen from Figure 10e,f under the SEM amplification of 1000 times, the wear surface of untreated cemented carbide samples was relatively rough, with more groove marks and obvious shedding of WC particles. After DCT, the wear surface was relatively flat and the shedding of WC particles was reduced on a large scale.

### 3.3. Electrochemical Corrosion

**Figure 11:**
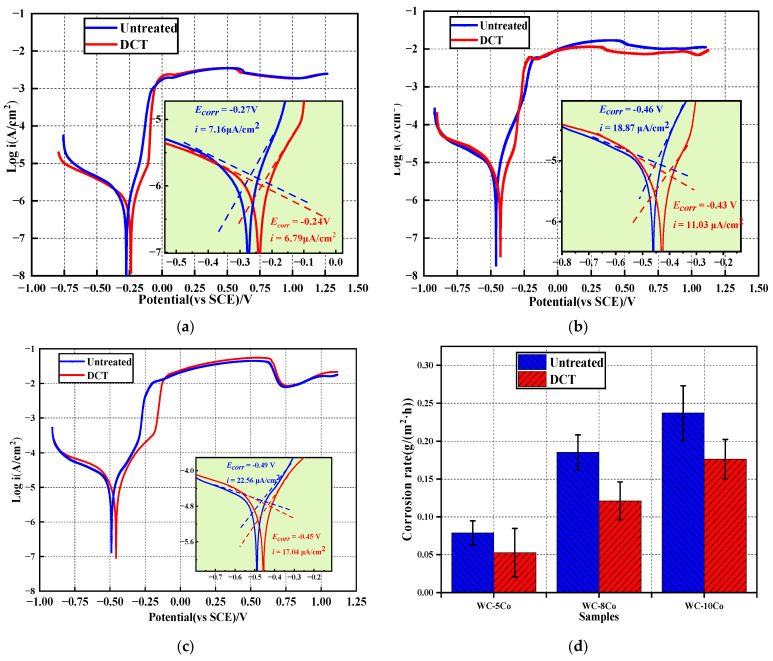
The polarization curves and corrosion rate of cemented carbide with untreated and DCT. (**a**) the polarization curve of WC-5Co with untreated and DCT; (**b**) the polarization curve of WC-8Co with untreated and DCT; (**c**) the polarization curve of WC-10Co with untreated and DCT; (**d**) the corrosion rate of cemented carbide with untreated and DCT.

Figure 11 shows the polarization curve of WC-Co cemented carbide (untreated and after DCT samples) with different Co contents in salt solution (3.5 wt% NaCl). Table 4 shows the corrosion potential and corrosion current density. As can be seen from Figure 10a–d, there was a small passivation area near +500 mV on the anodic polarization curve of WC-5Co carbide, and the corrosion current decreases slightly and then increases. WC-10Co had an obvious passivation zone near +675 mV, and the corrosion current continues to increase after a slight decrease. With the increase in Co content, the self-corrosion potential of cemented carbide tends to move in a negative direction. The more positive the self-corrosion potential and the smaller the corrosion current, the better the thermodynamic stability of cemented carbide under specific conditions and the slower the corrosion rate [40,41,42]. As can be seen from Table 4, the self-corrosion potential of WC-5Co and WC-10Co in 3.5% NaCl salt solution showed a positive shift, and the corrosion current density also decreased after DCT. Compared with the untreated cemented carbides, the growth rates of corrosion potential of WC-5Co, WC-8Co, and WC-10Co were 7.61%, 7.38%, and 9.11%, respectively, and the decrease rates of corrosion current density were 5.24%, 41.58%, and 24.47%, respectively.

The corrosion rate of metal materials can be expressed by the corrosion current density or corrosion rate. The corrosion current density and corrosion rate can be converted by Faraday’s theorem, and the corrosion rate is calculated according to Equation (4).

(4) $$v=\frac{Mi}{nF}=3.73\times 10^{-4}\frac{Mi}{n}({g/m}^{2}h)$$

where M is the atomic weight; i is the corrosion current density (μA/cm^2^); n is the valence of a metal; F is Faraday’s constant; ρ is the density of the material (g/cm^3^).

According to Equation (4), the corrosion rate was positively correlated with the corrosion current density, and the corrosion process was mainly caused by the corrosion of the bonding phase. The calculation of the corrosion rate of cemented carbide with different Co content is shown in Figure 10d. The DCT reduces the corrosion rate of cemented carbide and improves its corrosion resistance.

**Figure 12:**
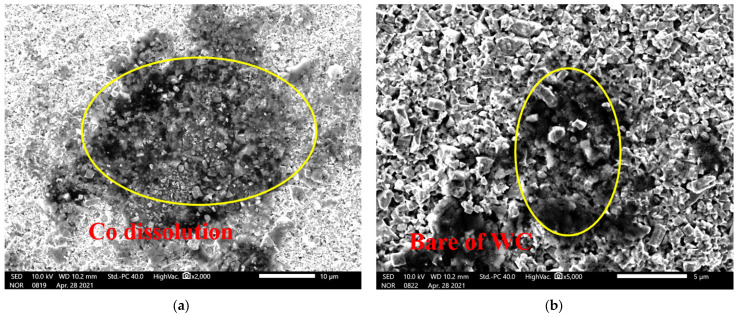
The SEM image of electrochemical corrosion of cemented carbide. (**a**) the Co dissolution in cemented carbide; (**b**) the WC particles bared in cemented carbide.

Figure 12 is the microstructure of the electrochemical corrosion surface of WC-Co. The corroded surface of the alloy was properly cleaned to remove the corrosive medium and loose surface attachment. As can be seen from Figure 12a, after the corrosion of the NaCl medium, the Co phase on the outer surface of the alloy was corroded and dissolved, but the WC grain remained intact, indicating that the corrosion of the alloy was mainly caused by the dissolution and corrosion of the bonding phase. It can be seen from Figure 12b that all the binder phases on the alloy surface were corroded and dissolved, while the hard-phase WC grains were in an “isolated” state due to the dissolution of the binder phase, and the corrosion further corrodes the interior of the cemented carbide matrix.

### 3.4. Mechanism Analysis

#### 3.4.1. Wear Mechanism Analysis

**Figure 13:**
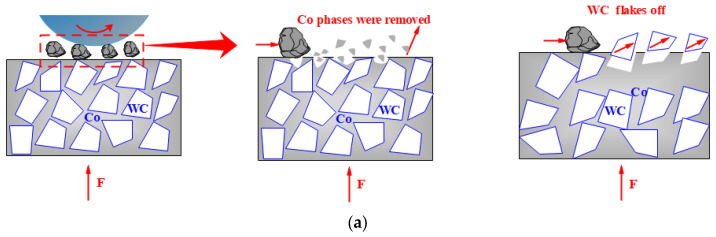

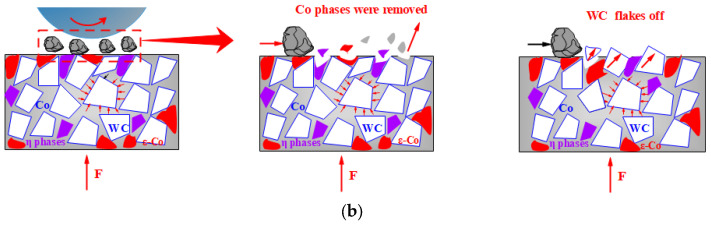
The mechanism of wear resistance of cemented carbide with untreated and DCT. (**a**) the mechanism of wear resistance of cemented carbide with untreated; (**b**) the mechanism of strengthening wear resistance of cemented carbide with DCT.

The strengthening mechanism of cryogenic treatment on the wear resistance of cemented carbide is shown in Figure 13: for untreated cemented carbide, in the initial stage of wear, the bonding phase Co within a certain depth range of the material contact surface was subjected to plastic deformation and micro-abrasive wear under the action of friction, and the surface Co was extruded from the WC grains. With the loss of binder Co, the surface integrity of the cemented carbide is damaged, the WC framework becomes unstable, and cracks occur among WC grains, so that some WC particles begin to pull out from the surface of the matrix [43,44,45,46]. The DCT can increase the residual compressive stress of the WC particles, and at the same time promote the precipitation of the η phase and the phase transition of Co in the cemented carbide, which leads to the increase in the residual compressive stress and densification degree of the surface of the cemented carbide, and increases the difficulty of the Co de-emission and the dislodgement of the WC particles [47,48]. Therefore, compared with the untreated cemented carbide, the wear surface of the deep-cryogenic-treated cemented carbide is smooth and has fewer abrasive marks.

#### 3.4.2. Corrosion Mechanism Analysis

The metal cobalt in cemented carbide was a kind of easy corrosion material; it was not only easy to dissolve in various kinds of strong acid, but at the same time, the weak acid medium of acetic acid also had a certain corrosive effect on it. Also, WC and Co have different electrode potential: with carbide immersion in the electrolyte solution, adjacent WC and Co will constitute the primary battery to result in corrosion of the cemented carbide.

Compared with the WC, the Co was prone to oxidation reaction, electron loss, and formation of ions into the solution [47,48,49]. Co^2+^ will coordinate with hydroxyl groups in H_2_O to form Co(OH)_2_ oxide film, covering the surface of the alloy:

(5) $$\mathrm{Co}-2e^{-}+2{\mathrm{OH}}^{-}\to {\mathrm{Co}(\mathrm{OH})}_{2}$$

**Figure 14:**
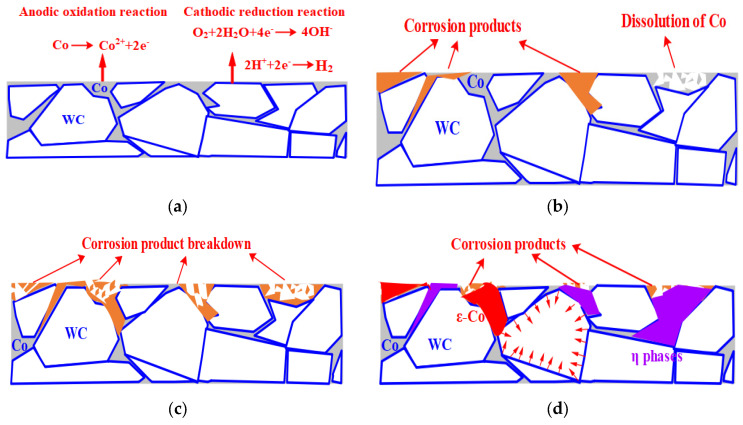
The corrosion reaction principles and strengthening mechanism of corrosion resistance of cemented carbide by DCT. (**a**) the electrode reaction; (**b**) the Co dissolution and formation of corrosion products; (**c**) the breakdown of corrosion products; (**d**) the strengthening mechanism of corrosion resistance of cemented carbide by DCT.

The formation of oxide film can reduce the diffusion of oxygen molecules into the alloy and inhibit the further corrosion of the alloy. With the further increase in anode potential, the oxide film was broken down, eventually leading to the leaching of Co within a certain thickness range on the alloy surface, and the corrosion was further intensified [48,49]. The specific corrosion reaction principle is shown in Figure 14a–c. Compared with WC-5Co, with the increase in Co content in cemented carbide, the more negative the self-corrosion potential of WC-10Co was, the greater the corrosion current density was.

The self-corrosion potential of WC-5Co, WC-8Co, and WC-10Co tended to move forward after DCT, and the corrosion current density also decreased slightly, indicating that DCT delayed the corrosion process to a certain extent. The specific corrosion reaction mechanism is shown in Figure 14d, which may be that DCT increases the compressive stress of WC on the cemented carbide surface. Moreover, there was precipitation of the η phase and Co phase transition after DCT, which improves the surface densification of cemented carbides, postpones the diffusion of ions in the corrosion process, inhibits the stress corrosion of materials, and improves the corrosion resistance of cemented carbides [39,40].

## 4. Conclusions

Through the wear resistance experiment and electrochemical corrosion experiment of cemented carbides, both untreated and after DCT, the influence of DCT on the wear resistance, corrosion resistance, and microstructure of WC-Co cemented carbides is explored, and the following conclusions are drawn:

(1) The DCT can reduce the wear rate of cemented carbide: the wear rate of WC-5Co, WC-8Co, and WC-10Co can be reduced by 14.71%, 37.25%, and 41.01% after DCT, respectively.
(2) The DCT can improve the corrosion resistance of cemented carbide: compared with the untreated cemented carbides, the growth rates of corrosion potential of WC-5Co, WC-8Co, and WC-10Co were 7.61%, 7.38%, and 9.11%, respectively, and the decrease rates of corrosion current density were 5.24%, 41.58%, and 24.47%, respectively.
(3) The wear patterns of cemented carbides are mainly the extrusion deformation of Co and the shedding of hard-phase WC during the wear process. The reason for the electrochemical corrosion of cemented carbide is that binder Co leached in the electrolyte solution, which aggravates the further corrosion of cemented carbide.
(4) The DCT promotes the precipitation of the η-phase, the transformation of α-Co to ε- Co, and the increase in WC residual compressive stress in cemented carbide. The densification and homogenization caused by the precipitation of the η-phase and the increase in WC residual compressive stress after DCT are the main reasons for the enhancement of wear and corrosion resistance of cemented carbide.

## Figures and Tables

**Table 1:** The composition and basic mechanical properties of cemented carbide.

Raw Powders	Purity (wt. %)	Average Particle Size (μm)	Oxygen Content (wt. %)	Carbon Content (wt. %)	Density (g/cm^3^)
WC	>99.5	0.6–1.0	<0.23	6.13	15.63
Co	>99.9	0.5	<0.09	-	7.78
VC	>99.0	2	<0.2	-	5.78
TaC	>99.0	1.5	<0.2	6.18	14.57

**Table 2:** Wear test conditions of cemented carbide.

Power (kw)	Voltage (V)	Diameter of Grinding Wheel (mm)	Linear Speed (m/s)	Rotate Speed (r/min)	Pressure (N)	Feed Speed (mm/s)
1.5	30	80	25	3750	30	2.5

**Table 3:** The residual compressive stress of cemented carbide.

Samples	WC-5Co (MPa)	WC-8Co (MPa)	WC-10Co (MPa)
Untreated	−346 ± 24	−406 ± 28	−489 ± 14
DCT	−637 ± 33	−777 ± 31	−807 ± 23

**Table 4:** The corrosion potential and corrosion current density.

Samples	Untreated				DCT			
	$E_{corr}$ (V)		i (μA/cm^2^)		$E_{corr}$ (V)		i (μA/cm^2^)	
WC-5Co	−0.24	−0.27	6.06	7.16	−0.32	−0.24	7.04	6.79
	−0.28		7.23		−0.17		6.48	
	−0.29		8.19		−0.23		6.85	
WC-8Co	−0.47	−0.46	19.53	18.87	−0.40	−0.43	10.42	11.03
	−0.39		16.91		−0.43		11.69	
	−0.50		20.17		−0.46		10.98	
WC-10Co	−0.53	−0.49	21.29	22.56	−0.48	−0.45	15.73	17.04
	−0.51		21.53		−0.40		17.56	
	−0.46		24.86		−0.47		17.83	

## Data Availability

Data are contained within the article.
